# Development of Oral In Situ Gelling Liquid Formulations of Garcinia Extract for Treating Obesity

**DOI:** 10.3390/gels9080660

**Published:** 2023-08-16

**Authors:** Kantiya Fungfoung, Rachanida Praparatana, Ousanee Issarachot, Ruedeekorn Wiwattanapatapee

**Affiliations:** 1 Department of Pharmaceutical Technology, Faculty of Pharmaceutical Sciences, Prince of Songkla University, Hatyai 90112, Songkhla, Thailand; kantiya.1537@gmail.com; 2 Faculty of Medical Technology, Prince of Songkla University, Hatyai 90112, Songkhla, Thailand; rachanida.p@psu.ac.th; 3 Pharmacy Technician Department, Sirindhron College of Public Health of Suphanburi, Mueang Suphan Buri District 72000, Suphan Buri, Thailand; ousaneemu@gmail.com; 4 Phytomedicine and Pharmaceutical Biotechnology Excellence Center, Faculty of Pharmaceutical Sciences, Prince of Songkla University, Hatyai 90112, Songkhla, Thailand

**Keywords:** garcinia extract, hydroxycitric acid, gastroretentive drug delivery system, in situ gel, obesity

## Abstract

Novel in situ gelling liquid formulations incorporating garcinia extract were developed to achieve prolonged delivery of hydroxycitric acid (HCA), an active compound displaying anti-obesity function, following oral administration. The optimized formulation was composed of sodium alginate (1.5% *w*/*v*), hydroxypropyl methylcellulose (HPMC K100) (0.25% *w*/*v*), calcium carbonate (1% *w*/*v*) and garcinia extract (2% *w*/*v*). The formulation displayed rapid gelation in less than a minute on exposure to 0.1 N hydrochloric acid (pH 1.2) and remained afloat for more than 24 h. The formulations were capable of gradually releasing more than 80% of HCA load over 8 h, depending on the composition. The resulting gels exhibited high values of gel strength by texture analysis, suggesting they would offer resistance to breakdown under the action of stomach content movement. The optimized formulation loaded garcinia extract significantly reduced lipid accumulation in 3T3-L1 adipocyte cells and displayed moderate anti-inflammatory activity by inhibiting the production of nitric oxide (NO) in LPS-stimulated RAW 264.7 macrophage cells. These findings demonstrate that oral in situ gelling liquid formulations based on sodium alginate and HPMC K100 offer much potential for sustained delivery of HCA and other anti-obesity compounds.

## 1. Introduction

Overweight and obesity are among the most prevalent risk factors associated with a number of acute and chronic health problems including type II diabetes, hypertension, osteoarthritis, cancer, and cardiovascular disease. Obesity in turn is linked to various causes, including a person’s genotype, a sedentary lifestyle, and excessive food consumption [1,2]. At present, a few drugs are available in the market, including sibutramine and rimonabant, that function as appetite suppressants to reduce obesity. Other therapeutics such as orlistat are designed to prevent fat absorption. However, most products display limited efficacy, along with major adverse effects affecting poor tolerability and safety [3]. In 2010 A.D., the United States Food and Drug Administration withdrew sibutramine and fenfluramine. Hence, considerable effort has been directed toward finding alternative and improved therapeutics for reducing obesity, with the investigation of herbal medicines receiving significant attention [4].

*Garcinia atroviridis* Griff. Ex T. Anderson also known as garcinia, Malabar tamarind, belongs to the plant family of *Clusiaceae* and is distributed mainly in Southeast Asia [5]. The native name in Thailand is “Som-Khaek” [6,7]. The dried fruit rind of garcinia has been used extensively for centuries as a condiment and flavoring agent in culinary dishes. The leaves and fruit have also been exploited in traditional medicine to exploit their thermogenic properties and increase metabolic activity and aid digestion. The herbal preparations or natural medicines prepared from garcinia rinds have also been applied in the treatment of inflammatory ailments and bowel conditions [8,9,10].

**Figure 1:**
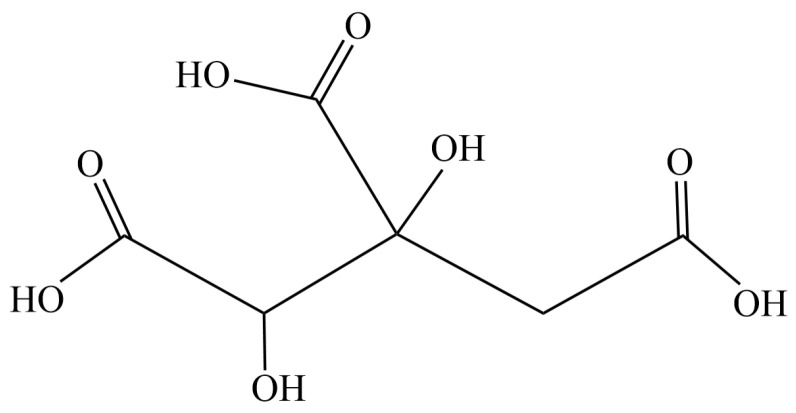
Structure of hydroxycitric acid (HCA).

Garcinia contains various phytochemicals including flavonoids and organic acids, among which hydroxycitric acid (HCA, C_6_H_8_O_8_, molecular weight 208.12 g/mol) (Figure 1) has been identified as an anti-obesity agent and a potential supplement for weight management [8]. HCA is water soluble and stable at room temperature in closed containers but incompatible with oxidizing agents. The *Garcinia atroviridis* extracts for commercial products are isolated from fruit rind and contain about 50% HCA.

Numerous studies have shown that consumption of HCA suppresses appetite and lowers the production of cholesterol and fatty acids. HCA reduces appetite, lipogenesis, and body weight by inhibiting an enzyme involved in fat storage in adipose tissue. HCA has been shown to be a competitive inhibitor of ATP citrate lyase [3,11,12]. Moreover, HCA increases hepatic glycogen synthesis, which helps produce a longer-lasting neurosignal from the liver to the brain, indicating satiety and thus prolonging appetite suppression [8,10].

HCA possesses significant advantages as a weight loss supplement due to its natural origin and safety profile. Studies both in human [13] and animal subjects [14] showed no serious side effects at a high dose of 5000 mg/kg, equivalent to 350 g/kg in humans [15]. Garcinia extract products currently available in the market are manufactured as conventional oral liquid, tablet, and capsule dosage forms, designed to be taken three times a day to deliver a dose of 1500–2800 mg [13,16,17]. However, the conventional oral dosage forms can result in poor bioavailability due to incomplete drug absorption, degradation in the gastrointestinal tract, and fast emptying times, resulting in poor compliance and inconsistent therapeutic efficacy.

Gastroretentive drug delivery systems (GRDDS) are designed to remain in the stomach for extended time periods following oral administration and gradually release active compounds in a controlled manner [18]. The reduced frequency of administration is intended to decrease drug dose and improve patient compliance. Various approaches have been investigated to prolong gastric residence time including density variation, the use of bioadhesive systems, and expandable and raft-like delivery devices that float on the stomach contents [19,20]. Oral in situ gelling systems have recently been investigated as a novel form of GRDDS. The formulations are administered as a low-viscosity liquid, and gel on exposure to stomach contents to prolong drug release [21,22]. The transition from liquid to gel may be induced by a shift in temperature in the case of thermo-gelling xyloglucan [23] or by the presence of cations in the case of gellan gum [24], sodium alginate and pectin hydrogels [22,23,25].

The purpose of this study was to develop an oral, liquid gastroretentive, in situ gelling formulation incorporating garcinia extract for sustained drug release in the stomach. Moreover, gelation of the dosage form and expansion on contact with gastric fluid was expected to augment the appetite suppression action of HCA. The anti-obesity activity of the formulation was assessed in vitro using a mouse 3T3-L1 cell line and the anti-inflammatory properties were investigated by using RAW 264.7 macrophage cells.

## 2. Results and Discussion

### 2.1. Physicochemical Characterization of In Situ Gelling Liquid Formulations

**Figure 2:**
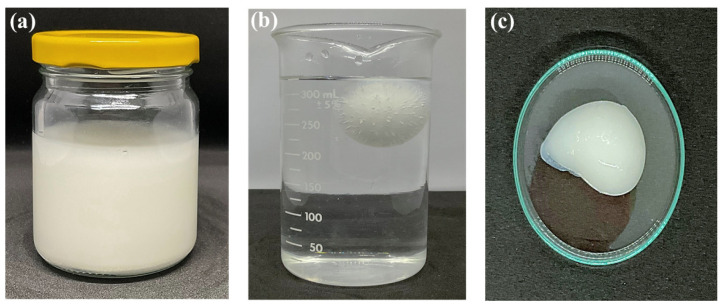
(**a**) Appearance of in situ gelling liquid formulation (G7), (**b**) floating behavior, and (**c**) formed gel.

All in situ gelling liquid formulations displayed uniform off-white or cream-colored appearance suggesting effective mixing of excipients and garcinia extract (Figure 2a). Substitution of the gelling agent, pectin, gellan gum, or xanthan gum resulted in incompatibility with the garcinia extract. The physicochemical properties of the formulation are presented in Figure 2 and Table 1.

#### 2.1.1. pH of Formulations

Aqueous solutions of sodium alginate are most stable in the pH range of 4 to 10 [26]. The pH of in situ gelling liquid formulations was found to lie in a narrow alkaline range between 9.3 and 9.8. The similarity of the alkalinity of the formulations was due to the addition of calcium carbonate in the same amount. All formulations revealed appropriate pH values that were suitable to maintain the formulations in a liquid state.

#### 2.1.2. Floating Behavior

Gelation of the liquid formulation and subsequent floating behavior of the formed gel on the gastric content will influence the amount and duration of HCA release. Gelation of the liquid formulations occurs by crosslinking of alginate by Ca^2+^ ions following the dissolution of the CaCO_3_ component. Additionally, the CO_2_ generated is entrapped within the hydrogel matrix resulting in floatation (Figure 2b). Gelation of the liquid formulations was rapid and occurred in less than a minute. Gelation times ranged between 14 and 51 sec and the floating lag time ranged between 9 and 31 min depending on the composition of the formulation (Table 1). The gelation time increased significantly upon raising the alginate concentration in the liquid formulation to 2% *w*/*v* (Table 1, formulations G1, G6, and G11). Moreover, the gelation time was highly dependent on the concentration of HPMC K100 in the formulation and almost doubled from 22 to 40 sec on increasing the concentration from 0.25 to 1% *w*/*v* (Table 1, formulations G7 to G10). This behavior suggests that the HPMC component hinders the dissolution of CaCO_3_ and/or access of the divalent ion to crosslinking sites on the alginate macromolecule [22]. The floating lag time of the gel was fairly constant (around 29–32 min) for formulations containing above 1% *w*/*v* alginate concentration and 0.5% *w*/*v* HPMC. Formulations containing lower concentrations floated in less than 20 min (G2, Table 1) and may reflect the time of CaCO_3_ and HCA release from the gel, which are initially present at high loading (1 and 2 g, respectively). All formed gels remained floating on the medium (0.1 N hydrochloric acid; pH 1.2) for more than 24 h, indicating the favorable gastroretentive properties of the formulation.

#### 2.1.3. Density

The density of gels formed on contact with 0.1 N hydrochloric acid (pH 1.2) for 30 min lay in a narrow range of 0.76 to 0.83 g/mL, indicating buoyancy in the medium at this particular time point as shown in Table 1. The density of all formulations was less than the density of 0.1 N hydrochloric acid: pH 1.2 (1.06 g/mL) [26]. Gel density tended to be affected slightly by the concentration of sodium alginate in the formulation. An increase from 1 and 1.5 to 2% *w*/*v* increased the gel density from 0.76 to 0.83 g/mL, while the increment of the concentration of HPMC K100 did not affect the density of the liquid in situ gel.

#### 2.1.4. Viscosity of In Situ Gelling Liquid Formulations

**Figure 3:**
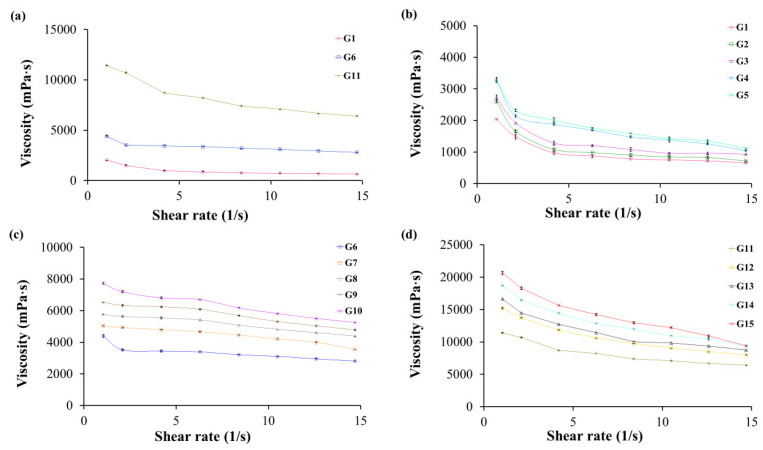
The viscosity of in situ gelling liquid formulations containing garcinia extract, (**a**) sodium alginate concentration 1, 1.5, and 2% *w*/*v*, (**b**) 1% *w*/*v* sodium alginate concentration, HPMC K100 concentration 0.25–1% *w*/*v*, (**c**) 1.5% *w*/*v* sodium alginate concentration, HPMC K100 concentration 0.25–1% *w*/*v*, (**d**) 2% *w*/*v* sodium alginate concentration, HPMC K100 concentration 0.25–1% *w*/*v*. Data represented as mean ± S.D. (*n* = 3).

The viscosity of in situ gelling liquid formulations gradually increased with increasing concentration of sodium alginate from 1 to 2% *w*/*v* and HPMC K100 from 0.25 to 1% *w*/*v* (Table 2) in line with the expected higher degree of polymer chain entanglement which contributes to a higher resistance to fluid flow. All the liquid formulations displayed pseudoplastic or shear thinning behavior signaled by a viscosity decrease in viscosity with increasing shear rate (Figure 3). This behavior is accepted as desirable for liquid formulations to ease the dispensing and dispersal of any sediment.

#### 2.1.5. Volume of the Formed Gel

The volume of gels formed on exposure of liquid formulations to 0.1 N hydrochloric acid (pH 1.2) was measured to evaluate their space-filling property in the stomach. Increasing the concentration of sodium alginate in the liquid formulation from 1 to 2% *w*/*v* significantly increased the volume of the formed gel from 30.17 to 45.33 mL. Moreover, increasing the HPMC K100 concentration from 0.25 to 1% *w*/*v* also significantly increased the gel volume (Table 2). The highest gel volume, about 45 mL, was produced by liquid formulations containing 2% *w*/*v* sodium alginate and 1% *w*/*v* HPMC K100 reflecting the weight of excipients and swelling of both sodium alginate and HPMC K100 in the 0.1 N hydrochloric acid medium (pH 1.2). The stomach capacity is approximately 572 ± 301.6 mL [27]. Thus, gels containing garcinia extract may prolong a satiated feeling due to a space-filling effect.

#### 2.1.6. Gel Strength

**Figure 4:**
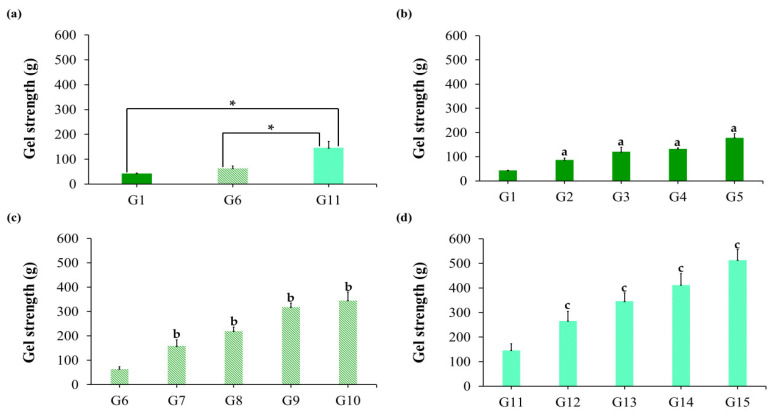
The gel strength of in situ gelling liquid formulations containing garcinia extract, (**a**) sodium alginate concentration 1, 1.5, and 2% *w*/*v*, (**b**) 1% *w*/*v* sodium alginate, HPMC K100 0.25–1% *w*/*v*, (**c**) 1.5% *w*/*v* sodium alginate, HPMC K100 0.25–1% *w*/*v*, (**d**) 2% *w*/*v* sodium alginate, HPMC K100 0.25–1% *w*/*v*. Data represented as mean ± S.D. (*n* = 3). (*) statistically significant differences were accepted at *p* values < 0.01. (^a^) *p* values < 0.01; statistical difference compared to G1 formulation (1% sodium alginate without HPMC). (^b^) *p* values < 0.01; statistical difference compared to G6 formulation (1.5% sodium alginate without HPMC). (^c^) *p* values < 0.01; statistical difference compared to G11 formulation (2% sodium alginate without HPMC).

Adequate gel strength is required to resist the breakdown of GRDDS as a result of movements of the stomach content. Increasing the concentration of sodium alginate (Table 2, Figure 4) resulted in significant increases in gel strength from 43.0, 63.4, and 146.1 g, respectively. This behavior may be explained by the increasing network strength resulting from the increased polymer content of the hydrogel and the higher density of carboxylic, hydroxyl groups that participate in ionic crosslinking with Ca^2+^ ions [28]. In addition, a major increase in gel strength was observed with increasing content of HPMC K100, suggesting a strong interaction between the polymer components [29].

#### 2.1.7. Entrapment Efficiency

According to Table 2, the very high percent HCA entrapment efficiency was obtained in the in situ gelling liquid formulations, ranging from 86 to 98% with no discernible effect of formulation composition. These results showed that HCA can be entrapped in the in situ gelling liquid formulations. Moreover, it indicated the compatibility between HCA and other ingredients in the formulation.

### 2.2. The Release Behavior of HCA from In Situ Gelling Liquid Formulations

**Figure 5:**
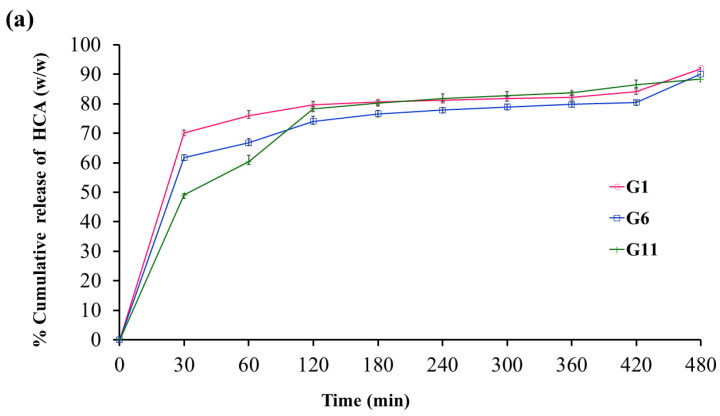

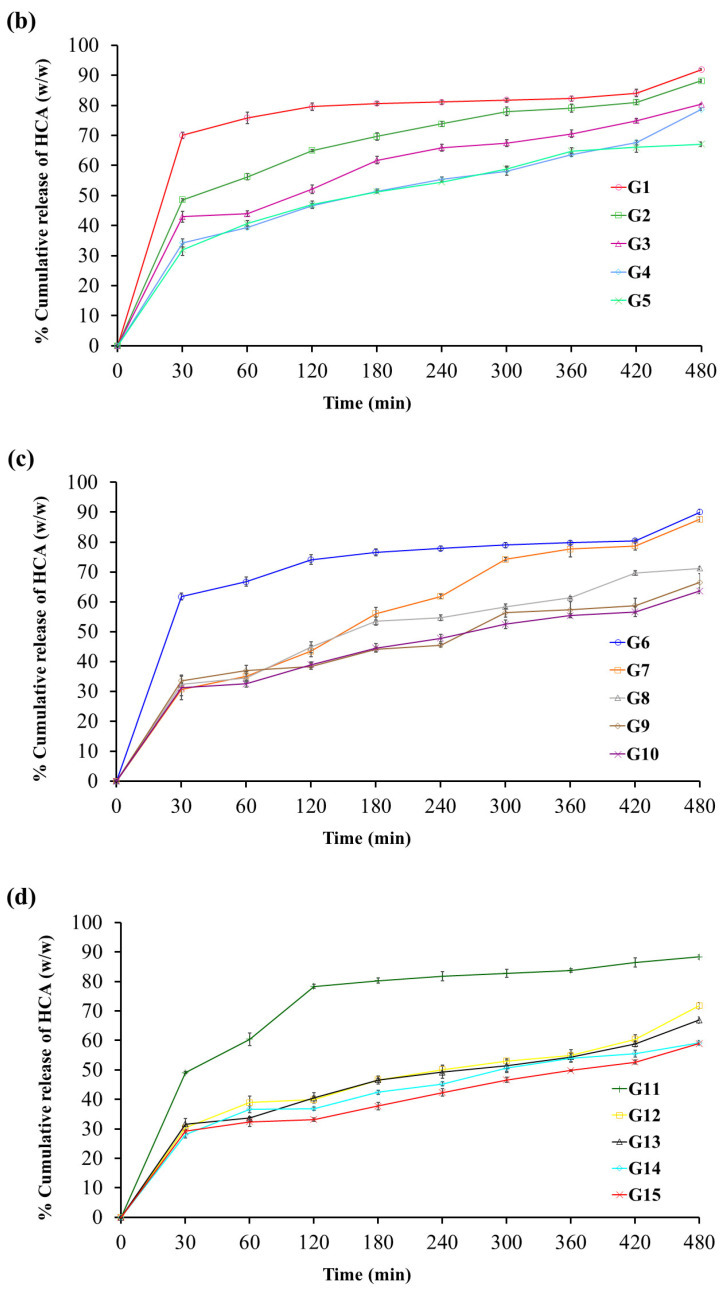
Release profiles of HCA from in situ gelling liquid formulations in 0.1 N hydrochloric acid (pH 1.2) at (**a**) sodium alginate, conc. 1, 1.5 and 2% *w*/*v*, (**b**) combination of 1% sodium alginate and 0.25–1% *w*/*v* HPMC K100, (**c**) combination of 1.5% sodium alginate and 0.25–1% *w*/*v* of HPMC K100, (**d**) combination of 2% sodium alginate and 0.25–1% *w*/*v* of HPMC K100. Data reported as mean ± S.D. (*n* = 3).

The release behavior of HCA from in situ gelling liquid formulations in 0.1 N hydrochloric acid (pH 1.2) is presented in Figure 5. The concentration of calcium carbonate was fixed at 1% *w*/*v* in order to avoid interference with the alginate structure [28], while the concentration of garcinia extract was set at 2 g. The release profiles of formulations containing only sodium alginate were similar (Figure 5a), featuring a burst effect at 30 min followed by a plateau phase extending from 2 to 8 h, during which cumulative HCA release was confined to around 80% of the initial content. Thus, highly efficient release occurs over a two-hour time period. The extent of burst release gradually decreased from 70 to 50% with increasing alginate content from 1 to 2% *w*/*v* and may be explained by a rise in crosslink density of the hydrogel which inhibits HCA diffusion and release [26,29,30].

The combination of sodium alginate (1% *w*/*v* conc.) and HPMC K100 at increasing concentrations from 0.25 to 1% *w*/*v* resulted in a significant decrease in the burst phase to 30–50% followed by gradual HCA release over 8 h, resulting in a maximum efficiency of around 80% (Figure 5b). This behavior highlights the role of HPMC in restricting the diffusion of HCA from the gel due to a high degree of polymer chain entanglement. Combining HPMC K100 with sodium alginate of 2% *w*/*v* concentration resulted in very similar release profiles; characterized by burst release of 30% and gradual release of up to 70% of the HCA content over 8 h (Figure 5d). The G7 formulation based on 1.5% *w*/*v* sodium alginate and 0.25% *w*/*v* HPMC K100 exhibited gradual and sustained release over 8 h, resulting in a highly efficient HCA release of around 90% (Figure 5c). In addition, the G7 formulation provided high gel strength and volume of formed gel (Table 2). Therefore, G7 was selected for in vitro bioactivity assay and stability studies. 

### 2.3. Release Kinetics and Mechanism Release of HCA from In Situ Gelling Liquid Formulation

The cumulative drug release of HCA incorporated into the liquid in situ gel formulation was found to be 87.67 ± 0.85% within 8 h. Fitting of the release data obtained for the G7 formulation with various kinetic models (zero-order, first-order, Higuchi, Hixson–Crowell, Korsmeyer–Peppas, and Weibull) indicated that Korsmeyer–Peppas model best represented the release behavior (Table 3). The correlation coefficient (R^2^) was 0.9900 that close to 1 and the *n* value was less than 0.45, indicating that the release mechanism was controlled by the Fickian diffusion of hydrophilic HCA through the hydrogel network. These patterns could be explained by diffusion and erosion mechanisms. In addition, the exponent b value calculated from the Weibull model was less than 0.75, indicating Fickian diffusion of the HCA from the polymeric network in the gel structure [31,32,33].

### 2.4. Stability Studies of In Situ Gelling Liquid Formulation G7

The methods section states that the physical and chemical stability of selected liquid formulations were tested at intermediate conditions (30 ± 2 °C, 65% ± 5% relative humidity; RH) and at accelerated conditions (45 ± 2 °C, 75% ± 5% RH). Furthermore, stability studies at 4 °C in the refrigerator were evaluated.

The results of stability testing of the in situ gelling liquid formulation G7 at 4 °C are presented in Table 4. No significant changes in visual appearance, pH, viscosity, gelation time, floating lag time, duration of floating, and gel strength were measured following storage in comparison with freshly prepared samples. Additionally, almost 100% of the HCA content remained stable following storage at 4 °C for 6 months. These results demonstrate that in situ gelling liquid formulations of garcinia extract may be stored in refrigerator settings for extended time periods, which simplifies storage logistics. In comparison, formulation G7 was found to have coagulated and was incapable of gelation after storage at 30 °C and 45 °C, respectively, for 2 weeks.

### 2.5. Biological Assay of In Situ Gelling Liquid Formulations of Garcinia Extract (G7)

#### 2.5.1. Effect on Cell Viability

**Figure 6:**
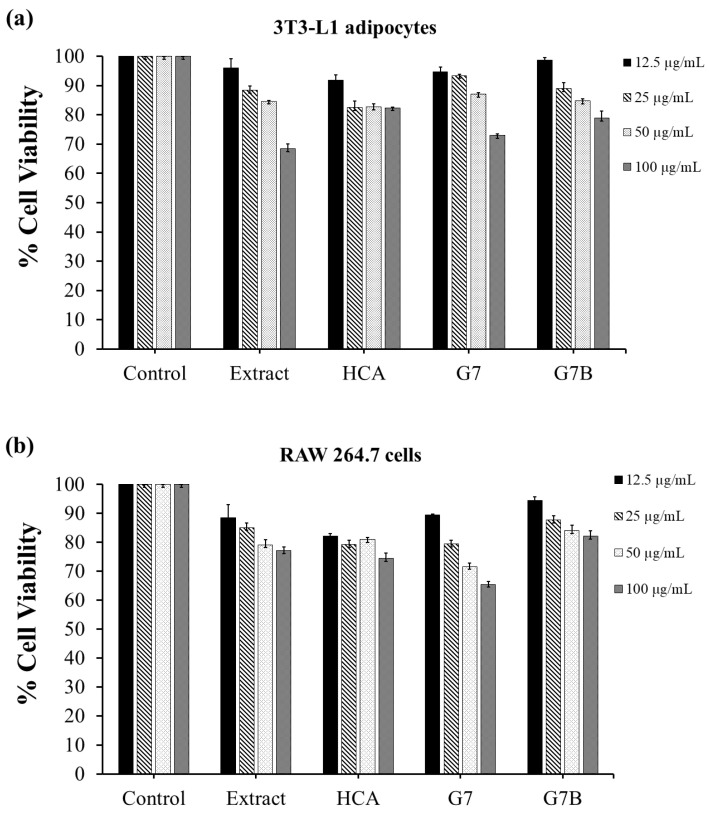
(**a**) Cell viability of 3T3-L1 adipocytes, (**b**) RAW 264.7 cells following incubation with control, garcinia extract, HCA standard, G7 formulation, G7 blank (G7B) for 24 h. Results expressed as a percentage of the control.

The effect of in situ gelling liquid formulations of garcinia extract (G7) on 3T3-L1 cells (used as a model of adipocyte differentiation and lipid accumulation) and RAW 264.7 macrophage cells (used as a model of anti-inflammation) is presented in Figure 6. The cytotoxicity of HCA standard, garcinia extract, G7 formulation, and G7 blank (G7B) toward both cell types was assessed 24 h post-treatment. The MTT assay showed that a 100 μg/mL dose of unformulated garcinia extract and G7 formulation reduces the viability of both cells, but cell viability remained above 80% at lower concentrations of extract (50, 25, 12.5 μg/mL) indicating no significant toxicity. Thus, a concentration of 50 μg/mL samples and below were selected for subsequent experiments. 

#### 2.5.2. Assay of Anti-Obesity Activity

**Figure 7:**
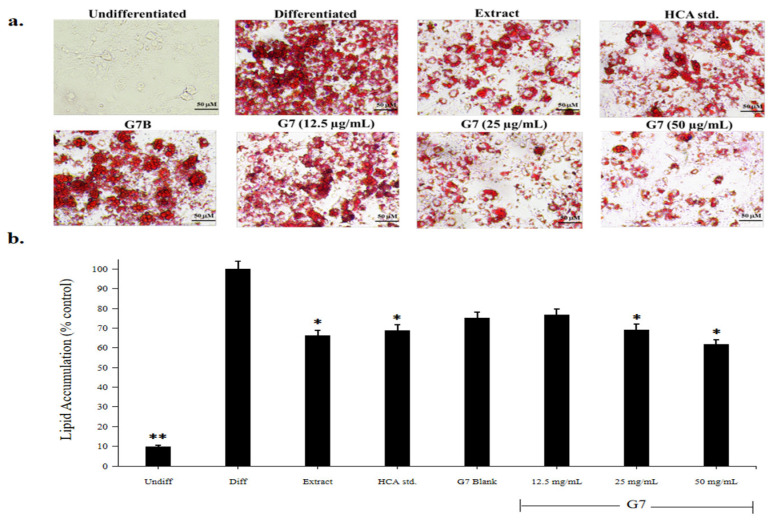
Lipid accumulation in 3T3-L1 adipocytes following treatment with 50 µg/mL garcinia extract, HCA standard, G7 blank (G7B), and various concentrations of G7 formulation (12.5, 25, 50 µg/mL) on Day 10. Culture media treatment was used as a negative control (undiff). (**a**) cellular lipid content revealed by Oil Red O Staining, (**b**) lipid accumulation quantified by UV absorbance measurement at 520 nm. Difference symbols (*, **) indicate statistical difference (*p* < 0.05).

Differentiation of preadipocytes into mature adipocytes occurs in response to the body’s need to store lipids due to changes in nutrition and inflammatory markers [34]. Preadipocytes were induced to differentiate in the presence or absence of garcinia extract, HCA standard, G7 formulation, and G7 blank for 10 days to investigate their effect on adipogenesis. Oil Red O staining (Figure 7a) revealed that preadipocytes (negative control) do not show lipid accumulation while differentiated cells or mature adipocytes (positive control) exhibited intense staining due to lipid presence. Exposure of preadipocytes to test samples (garcinia extract, HCA standard, G7 formulation, and G7 blank) resulted in lipid accumulation as revealed by Oil Red O staining [35]. Quantification of lipid presence by UV absorbance measurements showed that garcinia extracts at 50 μg/mL dose significantly reduced lipid accumulation by 3T3-L1 adipocytes to around 35% compared with the control (Figure 7b). The in situ gelling formulation of garcinia extract (G7) at the same concentration that delivered the HCA content of 22 µg/mL reduced lipid accumulation to a similar degree and dose-dependent activity was apparent over the range of 12.5 to 50 µg/mL.

Garcinia extract is commonly regarded as an herbal supplement that has good anti-obesity activity. Several studies have reported that HCA is a major bioactive constituent involved in lipid metabolism. Our results support these conclusions and demonstrate that HCA and the developed in situ gelling formulation could effectively inhibit adipogenesis in 3T3-L1 cells (Figure 7b).

#### 2.5.3. Assay of Anti-Inflammatory Activity

Obesity is associated with the overproduction of various cytokines, including IL-6, IL-8, TNF-α, and nitric oxide (NO), leading to chronic inflammation and the development of insulin resistance [2]. The level of NO was measured as nitrite concentration in the culture medium using Griess reagent. NO is one of the major inflammatory mediators produced by macrophages for eliminating pathogens. However, the accurate mechanisms and causes of inflammation in obesity are not clearly clarified. Lipopolysaccharide (LPS), a component of the cell wall of Gram-negative bacteria, is a strong activator of macrophage activity and the production of inflammatory cytokines. Garcinia extract, HCA standard, G7 formulation, and blank G7 formulation slightly inhibited NO production in LPS-induced RAW 264.7 cells and exhibited only moderate anti-inflammatory activity through this particular mechanism, compared with indomethacin as shown in Table 5. 

## 3. Conclusions

Novel GRDDS based on in situ gelling liquid formulations were developed for sustained oral delivery of HCA, a natural, anti-obesity compound. The formulations based on sodium alginate and HPMC gelled in less than a minute on contacting 0.1 N hydrochloric acid (pH 1.2). They remained afloat for over 24 h and gradually released over 80% of the bioactive content in 8 h. The gel carrier itself is expected to prolong a satiated feeling due to space-filling effects in the stomach. Taken together, these findings demonstrate that oral, in situ gelling liquid formulations offer much potential for sustained delivery of HCA and other anti-obesity compounds.

## 4. Materials and Methods

### 4.1. Materials 

Garcinia extract powder (~54.6% hydroxycitric acid) was provided by Tha Phra Chan Herb Co., Ltd. (Bangkok, Thailand). HCA standard compound (purity > 93.7%) (MW 208.12 g/mol) was obtained from ChromaDex^®^ Corp. (Los Angeles, CA, USA). Sodium alginate medium viscosity (viscosity of 2% *w/w* solution ~2000 cps; 25 °C) was purchased from High Science Ltd. (Songkhla, Thailand). Calcium carbonate was obtained from RCI Labscan (Bangkok, Thailand). Hydroxypropyl methyl cellulose (HPMC) Methocel K100LV was a gift from Colorcon Asia Pacific Pte Ltd. (Singapore) and sodium citrate was purchased from Sigma-Aldrich Co., Ltd. (St. Louis, MO, USA). All other reagents were of analytical grade.

3T3-L1 cells (CL-173^TM^, fibroblast cell line) and RAW 264.7 cells (TIB-71^TM^, murine macrophage cell line) were obtained from the American Type Culture Collection (ATCC; Manassas, VA, USA). High glucose Dulbecco’s modified eagle medium (DMEM), Roswell Park Memorial Institute 1640 medium (RPMI-1640), 3-(4,5-dimethyl-2-thiazolyl)-2,5-diphenyl-2H-tetrazolium bromide (MTT), penicillin–streptomycin, phosphate buffer saline (PBS; pH 7.4), trypsin EDTA 0.25%, and trypan blue solution were supplied by Gibco^®^ (Invitrogen, CA, USA). Fetal bovine serum (FBS) was purchased from Hyclone^TM^ (Cytiva, Bangkok, Thailand). Dimethylsulfoxide (DMSO) was obtained from Amresco^®^ (Solon, OH, USA). Lipopolysaccharide (LPS, from *Escherichia coli*). Isopropanol was sourced from Fisher Scientific International, Inc. (Hampton, VA, USA). Formalin was a gift from Songklanagarind Hospital (Songkhla, Thailand). Dexamethasone (DEX), 3-isobutyl-1-methylxanthanine (IBMX), insulin, indomethacin, Oil Red O solution 0.5% in isopropanol, and Griess reagent were purchased from Sigma-Aldrich (St. Louis, MO, USA). All other chemicals were of analytical or pharmaceutical grades.

### 4.2. Preparation of In Situ Gelling Liquid Formulations Loaded with Garcinia Extract

In situ gelling liquid formulations were prepared by combining sodium alginate as a gelling polymer and hydroxypropyl methylcellulose (HPMC) to achieve extended drug release behavior. The composition of the formulation is shown in Table 6. Briefly, sodium alginate was dissolved in 75 mL deionized water to produce 1, 1.5, and 2% *w*/*v* solutions, respectively. Sodium citrate (750 mg) was added, and the mixture was stirred until completely dissolved. HPMC K100 was added (except formulations G1, G6, and G11) to the alginate solution to obtain concentrations of 0.25, 0.5, 0.75, or 1% *w*/*v*, respectively, and stirring was continued to obtain clear solutions. Finally, calcium carbonate as a cross-linking agent (1 g) and garcinia extract (2 g) were added to each sodium alginate/HPMC blended solution, respectively. The liquid formulations were adjusted to 100 mL with deionized water and stored in sealed, light-resistant glass until further use.

### 4.3. Physicochemical Characterization of In Situ Gelling Liquid Formulations

#### 4.3.1. Physical Appearance and Measurement of pH

The physical appearance of garcinia extract liquid formulations was determined by visual observation of color, and homogeneity. Measurements of pH were obtained using a calibrated digital pH meter (FiveEasy F20, Mettler-Toledo GmBH, Zurich, Switzerland) at room temperature (25 ± 1 °C) in triplicate.

#### 4.3.2. Floating Behavior

The floating behavior of the liquid formulations was investigated by adding 30 mL to 900 mL of 0.1 N hydrochloric acid (pH 1.2) at 37 ± 0.5 °C. The time taken for the formed gel to float to the surface of the 0.1 N hydrochloric acid (pH 1.2) medium was designated “floating lag time” (FLT), while the duration of floating was recorded over 24 h. Each formulation was tested in triplicate.

#### 4.3.3. Gel Density

The density of gels formed by exposure of the liquid formulations to acidic medium was measured as follows. A measuring cylinder containing 75 mL of acidic medium (0.1 N hydrochloric acid; pH 1.2) was weighed (W_1_). Liquid formulation (5 mL) was gently added to the measuring cylinder to form a gel and the weight of the cylinder (g) was measured after 30 min (W_2_). The volume (mL) of the gel was recorded from the measuring cylinder scale (V). Gel density was calculated using equation [33]:

Density = W_2_ − W_1_/V (1)

#### 4.3.4. Viscosity of In Situ Gelling Liquid Formulations

The viscosity of in situ gelling liquid formulations was measured using a Brookfield digital viscometer (DV-III ultra, Middleboro, MA, USA) with spindle no.64 (LV4). The viscosity of the liquid formulations was measured under different angular velocities (5–70 rpm) at 25 ± 1 °C [36,37]. Rheograms were generated for each liquid formulation by plotting viscosity (mPas) versus shear rate (1/S). Each sample was measured in triplicate.

#### 4.3.5. Gel Strength

The gel strength resulting from in situ gelation of liquid formulations was evaluated by modifying the method of Nairy et al. [36]. A texture analyzer (TA. XT plus Texture Analyzer, Stable Micro Systems, Haslemere, UK) fitted with a 5 kg load cell was employed, and data were analyzed using Exponent software. Gel samples were placed in a Petrie dish and compressed using a 25 mm diameter cylindrical aluminum probe until the gel fractured. The compression speed was set at 1 mm/sec, over a distance of 8 mm, with an acquisition rate of 500 points per second, and a trigger force of 5 g was applied. The load–displacement graph was recorded and the maximum load (g) at gel rupture was reported as the gel strength. Each formulation was measured in triplicate and data were reported as mean ± S.D. (*n* = 3).

#### 4.3.6. Volume of the Formed Gel

The volume of the gel formed by in situ gelation of liquid formulations was evaluated using a measuring cylinder. Briefly, 30 mL of liquid formulation (equivalent to one dose) was poured carefully into 200 mL of medium (0.1 N hydrochloric acid; pH 1.2) contained in a 250 mL measuring cylinder. After that, the volume rising was observed on the scale bar of measuring cylinder and recorded. Each formulation was measured in triplicate.

#### 4.3.7. Determination of HCA Content of Liquid Formulations

The HCA loading of the liquid formulations (5 mL samples) was determined by extraction from deionized water (50 mL) contained in a volumetric flask. The mixture was sonicated for 30 min in an ultrasonic water bath (Crest Ultrasonic Corp., Ewing Township, NJ, USA), filtrated by using a 0.45 µm membrane filter (VertiPure™ PVDF(HL), Vertical Chromatography Co., Ltd., Bangkok, Thailand) and diluted to various concentrations in deionized water. The amount of HCA was analyzed by UV–visible spectrophotometry (UV-1900i, SHIMADZU Corporation, Kyoto, Japan) at 210 nm wavelength. The entrapment efficiency (EE) of HCA was calculated according to the following equation:

%Entrapment efficiency (EE) = (A/B) × 100(2)

where A is the measured amount of HCA in the formulation and B is the amount of HCA initially added to the formulation. Data were presented as the mean ± S.D. (*n* = 3).

#### 4.3.8. In Vitro Release of HCA from In Situ Gelling Liquid Formulations

Release of HCA from in situ gelling liquid formulations was evaluated using a USP Dissolution Apparatus II (paddle type) (PTWS 120D, Pharma Test Apparatebau AG, Hainburg, Germany). The release medium of 900 mL (0.1 N hydrochloric acid; pH 1.2) was maintained at 37 ± 0.5 °C. A paddle rotation speed of 50 rpm was applied in order to mimic the movement of stomach content. Liquid formulation (30 mL) was injected into the release medium using a disposable syringe and 5 mL was collected at predetermined time intervals (30, 60, 120, 180, 240, 300, 360, 420, and 480 min) and replaced by an equal volume of pre-warmed fresh medium. The release sample was filtered through a 0.45 µm membrane filter (VertiPure™ PVDF(HL)) and the concentration of HCA was measured by UV–visible spectrophotometry (UV-1900i, SHIMADZU Corporation, Kyoto, Japan) at 210 nm absorbance wavelength. The release profiles of HCA were plotted as percent cumulative release (%*w/w*) versus time (min). Each formulation was tested in triplicate and reported as mean ± S.D.

### 4.4. Release Kinetics

The release kinetics of HCA from the formulations were investigated by examining the data fit several mathematical models, namely zero-order, first-order, Higuchi model, Korsmeyer–Peppas model, Hixson–Crowell, and Weibull model, using the DDSolver program [38]. A coefficient of determination (R^2^) closest to 1 was considered to indicate the best fit of release data to a particular kinetic model.

### 4.5. Stability Studies

Stability testing of in situ gelling liquid formulations was performed according to the International Council for Harmonization of Technical Requirements for Pharmaceuticals for Human Use (ICH guidelines 2003) on the topic of Q1A (R2): stability testing of new drug substances and products. The physical and chemical stability of selected liquid formulations were tested at intermediate conditions (30 ± 2 °C, 65% ± 5% relative humidity; RH) and at accelerated conditions (45 ± 2 °C, 75% ± 5% RH) [32]. The optimized formulations were placed in tightly sealed glass containers and stored in a constant climate chamber (Memmert^®^ HPP260, New York, USA). Meanwhile, the stability of formulations was also studied at 4 ± 2 °C in a refrigerator. After 1, 3, and 6 months, the formulations were evaluated in terms of pH, gelation, floating behavior, density, viscosity, gel strength, and HCA content.

### 4.6. Biological Assay of In Situ Gelling Liquid Formulations of Garcinia Extract

#### 4.6.1. Cell Culture

Mouse 3T3-L1 preadipocytes were grown in DMEM, supplemented with 10% FBS and 1% penicillin–streptomycin at 37 °C under a humidified 5% CO_2_ atmosphere. The culture medium was changed every two days and cells were subcultured upon reaching 70% confluence. Murine macrophages (RAW 264.7 cells) were cultured in RPMI 1640 medium, supplemented with 10% FBS and 1% penicillin–streptomycin at 37 °C under a humidified 5% CO_2_ atmosphere. The cells were subcultured after reaching 100% confluence.

#### 4.6.2. Cell Differentiation

The 3T3-L1 fibroblasts were induced to differentiate into mature adipocytes by exposure to differentiation activation medium (D/A) as previously described [39]. In brief, differentiation was started by incubation of two days post-confluent cells with D/A media comprising 0.5 mM IBMX, 1 μM DEX, and 10 μg/mL insulin (defined as Day 0). On Day 2, the D/A medium was switched to the maintain medium (D/M, 10 μg/mL insulin in DMEM, high glucose) and refreshed every 2 days until cells were fully differentiated into adipocytes (Day 10–12).

#### 4.6.3. Cell Viability following Exposure to In Situ Gelling Liquid Formulations

The viability of both 3T3-L1 adipocytes [34,39,40] and RAW 264.7 cells [41] was measured by MTT reduction assay. The 3T3-L1 fibroblasts were seeded into 96-well plates at a density of 5000 cells/well and induced to fully differentiate as described above. The RAW 264.7 cells were seeded at a density of 50,000 cells/well in 96-well plates and grown overnight. 3T3-L1 adipocyte or RAW 264.7 cells were treated with various concentrations of standard, garcinia extract, optimized in situ gelling liquid formulation, and blank formulation for 24 h. The culture medium was removed, and the cells were incubated with 0.5 mg/mL MTT solution at 37 °C for 3 h. The supernatant was removed, and DMSO was added to dissolve the formazan crystals. The absorbance was measured at 570 nm using the microplate reader (Biotek model Power Wave X, Santa Clara, CA, USA), and cell viability was expressed as a percentage of the control value using the following equation:

(3) $$\mathrm{Cell}\;\mathrm{viability}\;\left[\%\right]=\frac{\mathrm{absorbance}\;\mathrm{of}\;\mathrm{sample}\;}{\mathrm{absorbance}\;\mathrm{of}\;\mathrm{control}}\times 100$$

#### 4.6.4. Assay of Anti-Obesity Activity

The inhibitory effect of standard, garcinia extract, optimized in situ gelling liquid formulation and blank formulation on lipid accumulation in 3T3-L1 cells was examined using Oil Red O staining. In brief, 3T3-L1 preadipocytes were seeded in 48-well plates at a density of 20,000 cells/well and induced to differentiate for 10 days in the absence or presence of standard, garcinia extract, optimized formulation, and blank formulation. Cells were washed with PBS and fixed with 10% formaldehyde for 60 min. The fixed cells were subsequently washed twice with 60% isopropanol and maintained at room temperature (RT) to dry completely. Oil Red O solution (200 µL) was added to each well plate and incubated at RT for 15 min. The cells were washed 4–5 times with deionized water to remove excess dye, prior to visualization of lipid staining under an inverted microscope [42,43,44]. The lipid accumulation was dissolved using 100% isopropanol and quantified by absorbance measurement at 520 nm using a microplate reader (Biotek model Power Wave X, Santa Clara, CA, USA). The level of lipid accumulation was expressed as inhibition percentage of adipocyte cells.

#### 4.6.5. Assay of Anti-Inflammatory Activity

The inhibitory effect of HCA on nitric oxide (NO) production in murine macrophages like RAW 264.7 cells was evaluated using a modification of the method reported by Kaewkroek et al., 2019 [41]. Cells were cultured in RPMI 1640 medium supplemented with sodium bicarbonate (0.1%) and glutamine 2 mM, penicillin G (100 units/mL), streptomycin (100 µg/mL), and 10% fetal calf serum. Cells were seeded at a density of 1 × 10^5^ cells /well in a 96-well plate and incubated at 37 °C under 5% CO_2_ for 1 h. The medium was removed and replaced with fresh medium containing 100 ng/mL of LPS and the test samples (standard, garcinia extract, optimized in situ gelling liquid formulation and blank formulation) at various concentrations. The cells were incubated for 24 h. Indomethacin was used as a positive control. The level of NO production was determined by assay of the amount of nitrile in the culture medium using Griess reagent [45]. The optical density was measured at 570 nm using a microplate reader (Biotek model Power Wave X, Santa Clara, CA, USA). The percentage inhibition of NO production was calculated using the following equation and IC_50_ values were determined (*n* = 5).

Inhibition (%) = [(A − B)/(A − C)] × 100(4)

where (A − C): NO_2_^−^ concentration (µM) [A: LPS (+), sample (−); B: LPS (+), sample (+); C: LPS (−), sample (−)].

### 4.7. Statistical Analysis

Data were presented as mean ± standard deviation (mean ± S.D.). The data were processed by Student’s *t*-test or one-way analysis of variance (ANOVA). *p* < 0.05 and *p* < 0.01 were regarded as statistically significant.

## Figures and Tables

**Table 1:** The effect of in situ gelling liquid formulation on pH, density, gelation time, and floating characteristics.

Formulation	pH	Density (g/mL)	Gelation Time (sec)	Floating Lag Time (min)
G1	9.58 ± 0.04	0.76 ± 0.03 ^x^	14.2 ± 3.7 ^x^	9.3 ± 0.8 ^y^
G2	9.81 ± 0.01 *	0.76 ± 0.01	18.3 ± 2.6 ^a^	19.6 ± 0.7 *
G3	9.81 ± 0.02 *	0.77 ± 0.01	19.6 ± 1.9 ^a^	20.7 ± 0.6 *
G4	9.86 ± 0.04 *	0.77 ± 0.01	24.6 ± 6.4 ^a^	22.9 ± 0.4 *
G5	9.86 ± 0.05 *	0.77 ± 0.01	31.5 ± 5.4 ^a^	26.6 ± 0.7 *
G6	9.74 ± 0.04 ^z^	0.78 ± 0.01	15.6 ± 4.7 ^x^	10.0 ± 1.3 ^y^
G7	9.41 ± 0.11 **	0.77 ± 0.03	21.7 ± 2.7 ^b^	26.1 ± 1.6 **
G8	9.26 ± 0.02 **	0.77 ± 0.01	25.7 ± 5.8 ^b^	29.2 ± 0.2 **
G9	9.30 ± 0.04 **	0.77 ± 0.02	35.6 ± 7.3 ^b^	29.7 ± 0.3 **
G10	9.29 ± 0.04 **	0.78 ± 0.03	40.0 ± 7.2 ^b^	30.0 ± 0.6 **
G11	9.73 ± 0.02 ^z^	0.80 ± 0.04	24.9 ± 3.1	15.4 ± 0.2
G12	9.43 ± 0.02 ***	0.81 ± 0.03	35.3 ± 5.8 ^c^	31.3 ± 0.2 ***
G13	9.44 ± 0.01 ***	0.82 ± 0.02	42.2 ± 9.5 ^c^	31.6 ± 0.4 ***
G14	9.55 ± 0.02 ***	0.82 ± 0.10	45.0 ± 5.2 ^c^	31.4 ± 1.1 ***
G15	9.55 ± 0.03 ***	0.83 ± 0.02	51.1 ± 7.6 ^c^	31.6 ± 0.4 ***

* *p* values < 0.01; statistically significant difference compared to G1 formulation (1% sodium alginate without HPMC). ** *p* values < 0.01; statistically significant difference compared to G6 formulation (1.5% sodium alginate without HPMC). ***^, y^ *p* values < 0.01; statistically significant difference compared to G11 formulation (2% sodium alginate without HPMC). Statistically significant differences compared to G1 formulation (1% sodium alginate without HPMC) were accepted *p* values < 0.05 (^a, z^). Statistically significant differences compared to G6 formulation (1.5% sodium alginate without HPMC) were accepted *p* values < 0.05 (^b^). Statistically significant differences compared to G11 formulation (2% sodium alginate) were accepted *p* values < 0.05 (^c, x^).

**Table 2:** The effect of in situ gelling liquid formulations on viscosity, gel volume, gel strength, and HCA entrapment efficiency.

Formulation	Viscosity at 60 rpm (mPas)	Volume of Formed Gel (mL)	Gel Strength (g)	HCA Entrapment Efficiency (%)
G1	716.5 ± 65.8	30.17 ± 0.29	43.0 ± 1.2	92.31 ± 2.66
G2	826.5 ± 23.1 *	30.83 ± 1.04	86.6 ± 8.3	92.13 ± 2.16
G3	959.8 ± 36.1 *	31.40 ± 1.22	120.3 ± 19.1	95.78 ± 1.49
G4	1256.9 ± 20.6 *	33.00 ± 0.87 *	132.5 ± 4.1	97.47 ± 0.40
G5	1346.4 ± 35.1 *	38.07 ± 0.12 *	177.9 ± 17.6	94.84 ± 0.14
G6	2952.7 ± 23.1 ^a^	31.67 ± 0.58 ^a^	63.4 ± 9.7	94.38 ± 3.86
G7	3995.8 ± 20.8 **	40.73 ± 1.10 **	158.6 ± 25.1	89.79 ± 1.53
G8	4599.0 ± 7.1 **	41.53 ± 0.81 **	219.4 ± 17.2	91.06 ± 0.51
G9	5032.3 ± 5.8 **	41.83 ± 1.26 **	318.6 ± 16.3	90.63 ± 0.57
G10	5505.5 ± 11.5 **	42.20 ± 0.35 **	344.9 ± 35.8	87.92 ± 0.22
G11	6688.6 ± 8.0 ^a^	36.33 ± 1.53 ^a^	146.1 ± 26.4	96.71 ± 0.34
G12	8498.2 ± 10.0 ***	41.50 ± 0.50 ***	265.6 ± 40.3	91.49 ± 0.10
G13	9351.3 ± 15.8 ***	43.17 ± 0.76 ***	345.8 ± 34.4	93.91 ± 0.12
G14	10,437.8 ± 129.9 ***	43.73 ± 1.03 ***	412.3 ± 46.9	89.90 ± 1.27
G15	10,916.0 ± 141.4 ***	45.33 ± 1.15 ***	513.5 ± 43.2	86.21 ± 0.38

* *p* values < 0.01; statistically significant difference compared to G1 formulation (1% sodium alginate without HPMC). ** *p* values < 0.01; statistically significant difference compared to G6 formulation (1.5% sodium alginate without HPMC). *** *p* values < 0.01; statistically significant difference compared to G11 formulation (2% sodium alginate without HPMC). Statistically significant differences when compared to G1 formulation were accepted *p* values < 0.05 (^a^).

**Table 3:** Release kinetic modeling of HCA from in situ gelling liquid formulation G7.

	Zero-Order (R^2^)	First-Order (R^2^)	Higuchi (R^2^)	Hixson–Crowell (R^2^)	Korsmeyer–Peppas		Weibull	
					R^2^	n	R^2^	b
G7	0.6616	0.9220	0.9812	0.8800	0.9900	0.427	0.9770	0.672

G7 formulation based on 1.5% *w*/*v* sodium alginate and 0.25% *w*/*v* HPMC K100.

**Table 4:** Physicochemical properties of in situ gelling liquid formulation (G7) following storage at 4 °C for 1, 3, and 6 months.

Test	Freshly Prepared	After 1 Month	After 3 Months	After 6 Months
pH	9.4 ± 0.1	9.4 ± 0.0	9.4 ± 0.1	9.2 ± 0.1
Gelation time (sec)	21.7 ± 2.7	22.3 ± 0.9	24.3 ± 1.5	24.2 ± 2.2
Floating lag time (min)	26.1 ± 1.6	27.0 ± 0.6	27.3 ± 0.9	26.9 ± 1.7
Duration of floating (h)	>24	>24	>24	>24
Density (g/mL)	0.77 ± 0.03	0.77 ± 0.03	0.78 ± 0.05	0.81 ± 0.03
Viscosity at 60 rpm (mPa⋅s)	3995.8 ± 20.9	3832.7 ± 15.8	3865.0 ± 10.0	3835.3 ± 61.6
Gel strength (g)	158.5 ± 25.1	156.5 ± 22.0	150.5 ± 17.4	148.7 ± 15.0

**Table 5:** Percentage of nitric oxide (NO) inhibition of various samples at 50 µg/mL on RAW 264.7 macrophage cells induced by lipopolysaccharide (LPS).

Samples	% Inhibition of Nitric Oxide Production
Indomethacin	66.93 ± 0.42
Garcinia extract	33.94 ± 0.74
HCA std.	29.55 ± 1.70
G7	39.44 ± 0.39
G7B	11.19 ± 0.85

**Table 6:** Composition of in situ gelling liquid formulations *.

Formulations	Concentration (%*w*/*v*)	
	Sodium Alginate	HPMC K100
G1	1	-
G2	1	0.25
G3	1	0.5
G4	1	0.75
G5	1	1
G6	1.5	-
G7	1.5	0.25
G8	1.5	0.5
G9	1.5	0.75
G10	1.5	1
G11	2	-
G12	2	0.25
G13	2	0.5
G14	2	0.75
G15	2	1

* Sodium citrate (750 mg), CaCO_3_ (1 g), and garcinia extract (2 g) added to each in situ gelling liquid formulation (100 mL).

## Data Availability

Not applicable.
